# Comparable Long-Term Outcomes of Cyclosporine and Infliximab in Patients With Steroid-Refractory Acute Severe Ulcerative Colitis: A Meta-Analysis

**DOI:** 10.3389/fmed.2019.00338

**Published:** 2020-01-21

**Authors:** Kata Szemes, Alexandra Soós, Péter Hegyi, Nelli Farkas, Adrienn Erős, Bálint Erőss, Emese Mezősi, Zsolt Szakács, Katalin Márta, Patrícia Sarlós

**Affiliations:** ^1^First Department of Medicine, Medical School, University of Pécs, Pécs, Hungary; ^2^Institute for Translational Medicine, Medical School, University of Pécs, Pécs, Hungary; ^3^Doctoral School of Clinical Medicine, University of Szeged, Szeged, Hungary; ^4^János Szentágothai Research Centre, University of Pécs, Pécs, Hungary; ^5^Momentum Gastroenterology Multidisciplinary Research Group, Hungarian Academy of Sciences, University of Szeged, Szeged, Hungary; ^6^Institute of Bioanalysis, Medical School, University of Pécs, Pécs, Hungary

**Keywords:** steroid-refractory, ulcerative colitis, cyclosporine, infliximab, colectomy, meta-analysis

## Abstract

**Background:** In steroid-refractory acute severe ulcerative colitis (ASUC), cyclosporine (CYS) or infliximab (IFX) may be considered as a second-line alternative to avoid colectomy. There are short-term data reported, but until now, there is no meta-analysis regarding long-term outcomes of CYS and IFX in patients with ASUC.

**Aim:** To compare long-term efficacy and safety of CYS and IFX in a meta-analysis.

**Methods:** Three electronic databases (PubMed, Embase, Cochrane Central Register of Controlled Trials) were searched for studies which compared CYS vs. IFX in adults with ASUC. Long-term colectomy-free rate from 1 to 10 years during CYS or IFX therapy was collected, last updated up to 22nd May 2019. Primary outcome was long-term colectomy-free rate, secondary outcomes were adverse events (AE), serious adverse events (SAE), and mortality. Long-term colectomy-free survival and safety measures were pooled with the random-effect model. Odds ratios (OR) with 95% confidence intervals (CI) were calculated.

**Results:** Data from 1,607 patients in 15 trials were extracted. In the first 3 years, pooled OR for colectomy-free survival was higher with IFX than with CYS (OR = 1.59, 95% CI: 1.11–2.29, *p* = 0.012; OR = 1.57, 95% CI: 1.14–2.18, *p* = 0.006; and OR = 1.75, 95% CI: 1.08–2.84, *p* = 0.024; at 1, 2, and 3 years, respectively). However, the significant difference remained undetected from the fourth year of follow-up and in subgroup of RCTs (OR = 1.35, 95% CI: 0.90–2.01, *p* = 0.143; OR = 1.41, 95% CI: 0.94–2.12, *p* = 0.096; and OR = 1.34, 95% CI: 0.89–2.00, *p* = 0.157; at 1, 2, and 3 years, respectively). No significant difference was detected regarding adverse events, serious adverse events and mortality between the groups. The neutral associations proved to be underpowered with trial sequential analysis.

**Conclusion:** However observational studies show IFX as a better choice, according to the RCTs, choosing either CYS or IFX as rescue therapy for ASUC, the long-term outcomes are not different, although further large RCTs are warranted.

## Introduction

Ulcerative colitis (UC) is a lifelong inflammatory bowel disease that causes a continuous mucosal inflammation of the colon and occurs periodically in patients' life. Acute severe ulcerative colitis (ASUC) is a life-threatening condition which requires hospitalization and occurs in about 25% of patients with UC (1). ASUC is defined as patients with bloody diarrhea ≥6/day and any signs of systemic toxicity [pulse > 90/min, temperature > 37.8°C, hemoglobin < 105 g/l, erythrocyte sedimentation rate (ESR) > 30 mm/h, or C-reactive protein (CRP) > 30 mg/l] (2). In the case of ASUC, intravenous (IV) corticosteroids are the mainstay of first-line treatment, but up to 40% of the cases are resistant to this therapeutic modality (3). In steroid-refractory cases, second-line therapy is advised to be introduced to avoid colectomy. Cyclosporine (CYS) and infliximab (IFX) are widely used as rescue therapies.

### Rationale

CYS is a calcineurin and cytochrome P450 inhibitor immunosuppressant blocking the transcription of cytokine genes (interleukin-2 and−4) in activated T cells, thereby reducing the inflammation in the intestine (4). In the 1990s, CYS was the first drug introduced as salvage therapy in steroid-refractory ASUC (5). In general, following 2 mg/kg/day IV CYS, 5 mg/kg oral CYS is recommended for up to 3 months as a bridge to an immunosuppressive agent [azathioprine (AZA) or 6-mercaptopurine (6-MP)] (6). Despite the fast response within 4–7 days and the reliable short-term effectiveness during CYS therapy, significant side effects may occur (7, 8). A close drug-level monitoring of CYS is required to avoid opportunistic infections, renal, vascular and neurological toxicity (9).

In the past 15 years, IFX, a chimeric IgG1 monoclonal antibody designed to bind tumor necrosis factor-alpha (TNFα) has become an alternative second-line therapeutic option in steroid-refractory ASUC (10). Regularly, a standard induction regimen of 5 mg/kg IFX is used, although recently accelerated dose intensification with 10 mg/kg IFX is often applied as well to counteract the increased intestinal clearance of IFX in ASUC (11). However, there is no data to support the benefit of 10 mg/kg. During IFX-linked immunosuppression, opportunistic infections, reactivation on latent tuberculosis or hepatitis may occur; therefore, careful screening is recommended before the initiation of IFX.

### Objectives and Research Question

In the treatment of steroid-refractory ASUC, two randomized controlled trials (RCTs) demonstrated equal short-term efficacy and safety of IFX and CYS (CYSIF, CONSTRUCT) (12, 13). These results were opposed by a previous meta-analysis of observational studies, where IFX was associated with significantly higher rates of treatment response and a lower 12 months colectomy-rate compared to that with CYS (14). A lately reported network meta-analysis with benefit-risk analysis also suggested that there is a rank order of efficacy for colectomy-free rates favoring IFX over CYS, although the difference between the treatments was small (15).

Since new studies have been released and long-term survival data have become available. Therefore, we aimed to summarize the currently available evidence on the long-term efficacy and safety of IFX and CYS in steroid-refractory ASUC.

## Methods

### Study Design, Participants, Interventions, and Comparators

This meta-analysis was reported in accordance with the Preferred Reporting Items for Systematic Reviews and Meta-Analysis (PRISMA) statement (Supplementary Table 1) (16). The protocol for this study was registered in the International Prospective Register of Systematic Reviews (PROSPERO) *a priori* under number CRD42018115035.

### Search Strategy

We searched MEDLINE via PubMed (http://www.ncbi.nlm.nih.gov/pubmed), Embase (https://www.embase.com) and Cochrane Central Register of Controlled Trials (CENTRAL) (http://www.cochranelibrary.com) databases from inception up to 22nd May 2019.

Our search followed the PICO concept. Studies discussed a population (P) of patients with steroid-refractory ASUC who received IFX (I) or CYS (C) as salvage therapy. The primary outcome (O) was long-term colectomy-free survival rate, defined as the follow-up period exceeding 12 months after therapy initiation. Secondary outcomes were adverse events (AE), serious adverse events (SAE) and mortality. AE and SAE were categorized in accordance with the definitions of the International Conference on Harmonization of Technical Requirements for Registration of Pharmaceuticals for Human use—Good Clinical Practice (ICH-GCP) consensus guidelines (17).

The following query combining Medical Subject Headings (MeSH) and free text terms were used'. (“colitis, ulcerative”[MeSH Terms] OR (“colitis”[All Fields] AND “ulcerative”[All Fields]) OR “ulcerative colitis”[All Fields] OR (“ulcerative”[All Fields] AND “colitis”[All Fields])) AND (“infliximab”[MeSH Terms] OR “infliximab”[All Fields]) AND (“cyclosporine”[MeSH Terms] OR “cyclosporine”[All Fields] OR “cyclosporin”[All Fields]) AND (“colectomy”[MeSH Terms] OR “colectomy”[All Fields]). We imposed only “English-language” and “human” filters on the search.

### Study Selection

After the database search, one author (KS) removed the overlapping records using a reference management software (EndNote X8, Clarivate Analytics, Philadelphia, PA, USA). Two investigators (KS and PS) independently screened titles, abstracts, and full-texts against the predefined eligibility criteria. Consensus involving a third party (PH) resolved discrepancies in each phase of selection.

We included any controlled studies (observational or experimental) that met the following criteria: (a) adult ASUC patients (aged ≥18 years) being refractory to IV or oral steroid treatment; (b) CYS and IFX was used as salvage therapy after 3–7 days of steroid treatment; (c) colectomy-free survival rate was assessed at 12 months or later; and (d) cytomegalovirus infection was not verified in the patients. There was no restriction for additional drugs used in UC treatment (e.g., AZA, 6-MP or methotrexate).

### Data Extraction, Quality Assessment

The following data were extracted from each study (Table 1): first author, year of publication, study type (prospective/retrospective; randomized/non-randomized), drug regimen, the number of patients, age, gender distribution, rate of extensive colitis, concomitant, and maintenance therapy, follow-up period and the definition of ASUC. Intention-to-treat data were extracted from RCTs. If numerical data on long-term colectomy-free survival were not reported (13, 23, 26, 31), we extracted data from the Kaplan-Meier curves by identifying the values on the axes “x” and “y” with a software [GetData Graph Digitizer] according to the method proposed by Guyot et al. (32). Data collection was accomplished by two authors independently (KS and PS). Discrepancies were resolved by consensus. In the case of any disagreement, a third author was involved to resolve conflicts (PH).

**Table 1:** Study characteristics.

**References**	**Drug regimen (number of patients)**	**Age (years)**	**Male (%)**	**Extensive colitis (%)**	**Concomitant medication**	**Maintenance therapy**	**Follow-up period**	**Definition of ASUC**
**RANDOMIZED CONTROLLED TRIALS**								
Laharie et al. (18)^a^	IFX (55): 5 mg/kg at 0, 2, 6 weeks	36 (26–51)^b^	28 (51%)	31 (55%)	AZA starting at day 7	AZA	7 years	Lichtiger score >10 + Mayo score
	CYS (60): 2 mg/kg/day IV for 1 week, then 4 mg oral for 3 months	39 (26–50)^b^	13 (22%)	34 (60%)	AZA starting at day 7	AZA		
Scimeca et al. (19)^a^^**^	IFX (17): 5 mg/kg at 0, 2, 6 weeks	39 ± 12^c^	not reported	13 (77%)	previous use: AZA/MP, steroid	AZA	1 year	Truelove and Witts score
	CYS (13): 5 mg/kg/day oral	39 ± 15^c^	not reported	11 (85%)	previous use: AZA/MP, steroid	AZA		
Williams et al. (13)**	IFX (135): 5 mg/kg at 0, 2, 6 weeks	39.3 ± 15.5^c^	89 (66%)	53 (39%)	AZA/6-MP started at week 4	AZA/6-MP + IFX	3 years	Truelove and Witts + Mayo score
	CYS (135): 2 mg/kg/day IV for 1 week, 5.5 mg/kg oral for 3 months	39.8 ± 15^c^	81 (60%)	62 (46%)	AZA/6-MP started at week 4	AZA/6-MP		
**OBSERVATIONAL STUDIES**								
Croft et al. (20)^a^	IFX (37): 5 mg/kg single-dose infusion	26 (20–43)^b^	15 (41%)	27 (73%)	AZA/6-MP/MTX	AZA/6-MP/MTX	1 year	Truelove and Witts score
	CYS (43): 4 mg/kg (1999–2003), 2 mg/kg (2003–2007), IV for 7 days, then oral for 3 months	28 (20–37)^b^	26 (60%)	32 (74%)	AZA/6-MP/MTX	AZA/6-MP/MTX		
Daperno et al. (21)^d^	IFX (6): 5 mg/kg at 0, 2 weeks	Not reported	Not reported	Not reported	Steroid	AZA	4 years	Truelove and Witts score
	CYS (15): oral 5 mg/kg/day	Not reported	Not reported	Not reported	Steroid	AZA		
Dean et al. (22)^d^	IFX (19): 5 mg/kg, max. 5 infusion	25 (16–85)^b^	11 (58%)	10 (53%)	AZA	AZA/6-MP/MTX	1 year	not reported
	CYS (19): 2 mg/k/day until response, then oral	31 (15–56)^b^	12 (39%)	9 (47%)	AZA	AZA		not reported
Duijvis et al. (23)^d^	IFX (22): 5 mg/kg IV at 0, 2, 6 weeks	35.5 ± 15.4^c^	14 (64%)	10 (45%)	AZA/6-MP/mesalazin	IFX	8 years	Mayo score
	CYS (33): 2 mg/kg/day IV until response, then oral for 3 months	37.7 ± 13.6^c^	17 (52%)	17 (52%)	AZA/6-MP/mesalazin	AZA/6-MP		
Kim et al. (24)^d^	IFX (33): 5 mg/kg IV at 0, 2, 6 weeks	44 (15–71)^b^	25 (76%)	12 (36%)	AZA	AZA + IFX	3 years	According to international criteria
	CYS (10): 2 mg/kg IV until response, then AZA	56 (22–72)^b^	3 (30%)	8 (80%)	AZA	AZA		
Mocciaro et al. (25)^d^	IFX (30): 5 mg/kg IV at 0, 2, 6 weeks	37 ± 16.6^c^	15 (50%)	20 (67%)	AZA	AZA	3 years	Truelove and Witts + Lichtiger score
	CYS (35): 2 mg/kg/day IV, if responded, switch to oral after 14 days (5 mg/kg)	34.9 ± 13.7^c^	15 (43%)	29 (83%)	AZA	AZA		
Naves et al. (26)^d^	IFX (30): 5 mg/kg IV at 0, 2, 6 weeks	38 (27–56)^b^	30 (100%)	21 (70%)	AZA/6-MP	IFX	6 years	Montreal severity score
	CYS (20): 2–4 mg/kg	42 (30–50)^b^	13 (65%)	14 (70%)	AZA	AZA		
Ordás et al. (27)^d^	IFX (131): 5 mg/kg IV at 0, 2, 6 weeks	40 (13–83)^b^	76 (58%)	91	AZA	AZA	5 years	According to international criteria
	CYS (377): 2–4 mg/kg, then 5–10 mg/kg oral	36 (9–83)^b^	217 (58%)	295	NA	AZA		
Protic et al. (28)^d^	IFX (54): 5 mg/kg IV at 0, 2, 6 weeks	39 (16–90)^b^	47 (87%)	49 (65%)	NA	IFX	1 year	Truelove and Witts + Lichtiger score
	CYS (38): 2–4 mg/kg IV for 7 days, then 5 mg/kg oral				AZA	AZA		
Radojcic et al. (29)^d^^**^	IFX (13): not reported	Not reported	Not reported	Not reported	Not reported	Not reported	1 year	Not reported
	CYS (15): not reported	Not reported	Not reported	Not reported	Not reported	Not reported		
Sjöberg et al. (30)^d^	IFX (49): 5 mg/kg single-dose infusion	38 (17–60)^b^	30 (61%)	42 (44%)	AZA/6-MP/5-ASA	AZA/6-MP	1 year	Truelove and Witts score
	CYS (43): 4 mg/kg for 7 days, then oral 4 mg for 18 weeks	32 (17–72)^b^	21 (49%)	30 (70%)	AZA/6-MP	AZA/6-MP		
Song et al. (31)^d^^**^	IFX (97): not reported	Not reported	Not reported	Not reported	Not reported	Not reported	10 years	Truelove and Witts + Mayo score
	CYS (23): not reported	Not reported	Not reported	Not reported	Not reported	Not reported		

a *Prospective study*.

b *Median + interquartile range (IQR)*.

c *Mean ± SD (standard deviation)*.

d *Retrospective study*.

ASUC, acute severe ulcerative colitis; IFX, infliximab; CYS, cyclosporine; AZA, azathioprine; 6-MP, 6-mercaptopurine; MTX, methotrexate; IV, intravenous; ^**^, abstract form only.

We assessed the risk of bias of observational studies using the Newcastle-Ottawa scale (NOS) (Table 2) (33). There is a reliable “star system” that has three broad perspectives to secure a simple tool for quality assessment: selection and comparability of the groups, and the ascertainment of the outcome. The quality of the included RCTs was assessed with the Cochrane Risk of Bias Tool along seven domains (34). After the assessment, low, high and unclear risks of bias were indicated with green, red and yellow symbols.

**Table 2:** Modified Newcastle-Ottawa Scale.

	**Newcastle-Ottawa scale items**	**High-quality items carrying a low risk of bias (green)**	**Low-quality items carrying a high (red) or an unknown (yellow) risk of bias**
Selection	Item 1: Representativeness of the initial study population—acute severe ulcerative colitis (ASUC)	Only patients with ASUC were included	Low: beside ASUC moderately severe UC cases were included. Unclear: no data on selection process.
	Item 2: Representativeness of the initial study population (ASUC)	Only patients with ASUC were included	Low: beside ASUC moderately severe UC cases were included. Unclear: no data on selection process.
	Item 3: Ascertainment of severity of ulcerative colitis	ASUCs was defined with objective scores (e.g., Lichtiger score, Mayo score)	Low: UC was defined with no objective scores Unclear: no data about objective severity score
	Item 4: Demonstration that outcome of interest was not present at start of study	The patients had no colectomy before and were treated with steroid as rescue therapy	Low: patients had any kind of colon resection before Unclear: no statement.
Comparability	Item 5: Study controls for age, sex	No significant difference was detected between patients treated with cyclosporine or infliximab regarding age.	Low: significant difference was detected between patients treated with cyclosporine or infliximab regarding age. Unclear: no comparison was performed based on age.
	Item 6: Study controls for extent of disease	No significant difference was detected between patients treated with cyclosporine or infliximab regarding extent of disease.	Low: significant difference was detected between patients treated with cyclosporine or infliximab regarding extent of disease. Unclear: no comparison was performed based on extent of disease.
Outcome	Item 7: Assessment of outcome	Colectomy-free survival rate or numbers of patients with colectomy were presented at least 1-year follow-up	Low: colectomy rate only available from the Kaplan-Meier curve Unclear: no statement
	Item 8: Adequacy of follow-up	At least 12 months follow-up period	Low: incomplete follow-up with explanations Unclear: incomplete follow-up without explanation of the loss.

### Data Analysis

Data on colectomy-free survival were extracted with IFX and CYS. Odds ratios (ORs) were calculated with 95% confidence intervals using the random effects model with the DerSimonian–Laird estimation (35). Results of the meta-analysis were displayed graphically using Forest plots. All analyses were two-tailed and *p* < 0.05 was considered as significant.

Subgroup analyses were performed to examine different effects in a 10 years interval. We carried out subgroup analyses only for the first 4 years based on the study design because data from RCTs were lacking for longer follow-up. Heterogeneity was tested by using the Cochrane's Q and the *I*^2^ statistics, where *I*^2^ = 100% × (Q – df)/Q and represents the magnitude of the heterogeneity (moderate: 30–60%, substantial: 50–90%, considerable: 75–100%) (34). All meta-analytical calculations were performed with Stata 15.1 data analysis and statistical software (Stata Corp LLC, College Station, TX, USA).

### Trial Sequential Analysis

**Figure 1:**
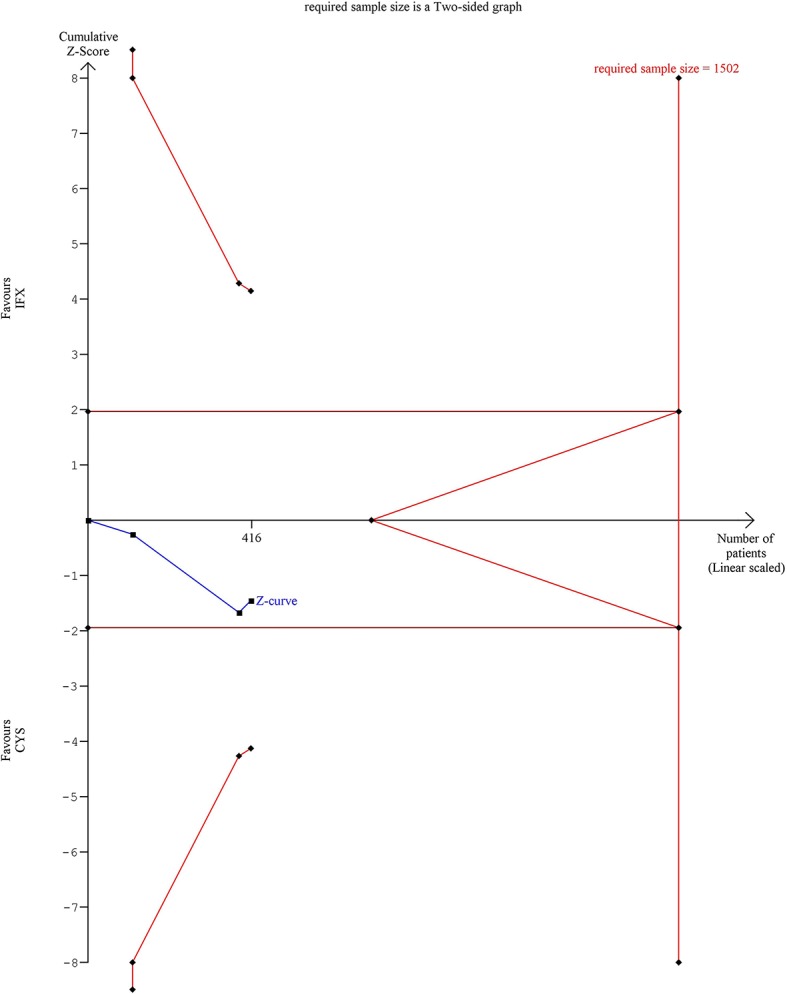
Results of the trial sequential analysis of the risks of 1 year colectomy-free rate. The required sample size of 1,502 patients was estimated using α = 0.05 (two-sided) and ß = 0.02 (power of 80%). Crossing of the constructed cumulative Z-curves (blue) and the two-sided *Z* = 1.96 provides a traditionally significant result. To obtain reliable evidence, crossing of the trial sequential monitoring boundaries (red) is needed. In the case of 1 year colectomy-free rate outcome, the cumulative Z-curve (blue) did not crossed the conventional boundary and neither the trial sequential monitoring boundary nor the required sample size line was surpassed.

**Figure 2:**
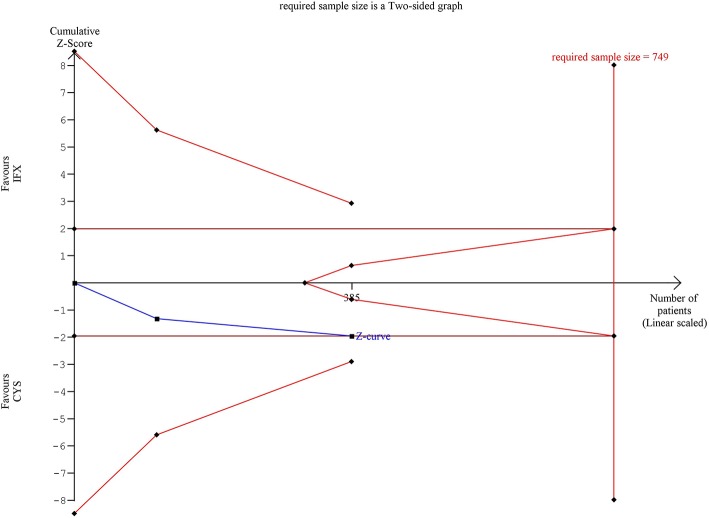
Results of the trial sequential analysis of the risks of adverse events. The required sample size was calculated with α = 0.05 (two-sided) and ß = 0.02 (power of 80%). Although cumulative Z-curve (blue) reached the conventional boundary, it did not cross through. Number of participants (385) did not reach the information size (749) and the cumulative Z-curve does not cross the monitoring boundary either.

**Figure 3:**
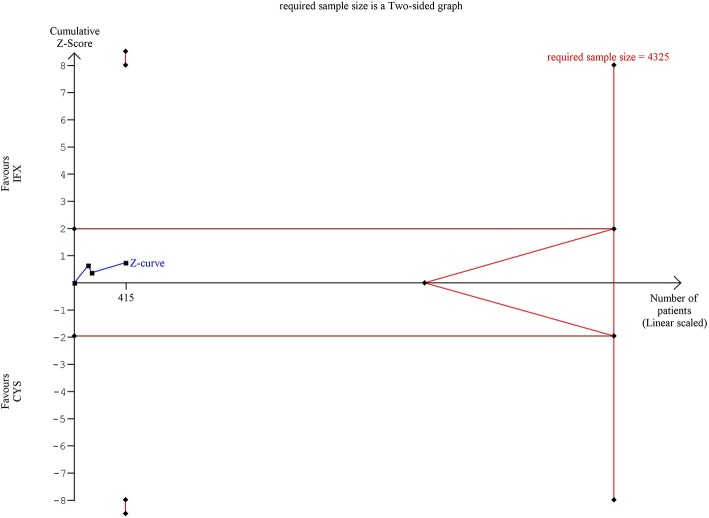
Results of the trial sequential analysis of the risks of serious adverse events. The required sample size was calculated using α = 0.05 (two-sided) and ß = 0.02 (power of 80%). The cumulative Z-curve (blue) did not cross the monitoring boundary (red) and not reached the required information size (4,325 patients). There is insufficient information about the evidence of significance.

Trial sequential analysis (TSA) was performed to assess the risk of type-I error and to estimate the required information size for an adequate statistical power if only RCTs were included (36). TSA was interpreted with an overall five percent of risk of type-I error (α = 0.05) and with a power of 80% (Figures 1–3). Crossing of the constructed cumulative Z-curves (blue) and the two-sided Z = 1.96 provides a traditionally significant result. To obtain reliable evidence, crossing of the trial sequential monitoring boundaries (red) is needed. We conducted TSA using Trial Sequential Analysis software 0.9 (Copenhagen Trials Unit, Denmark).

### Quality of Evidence

The GRADE system was constructed for the assessment of the quality of the evidence for the main outcomes in a review (37). The rating extends from very low to high quality, wherein RCTs starting from a high, non-randomized studies starting from a low quality of evidence. After the assessment of study design, outcomes were tested against five criteria including risk of bias, inconsistency, indirectness, imprecision and publication bias. Finally, the overall quality of the evidence for each outcome was graded as high, moderate, low or very low. Grading was performed independently by two of the authors (KS and PS) and disagreements were discussed by involving a third party (AE).

## Results

### Search Results

**Figure 4:**
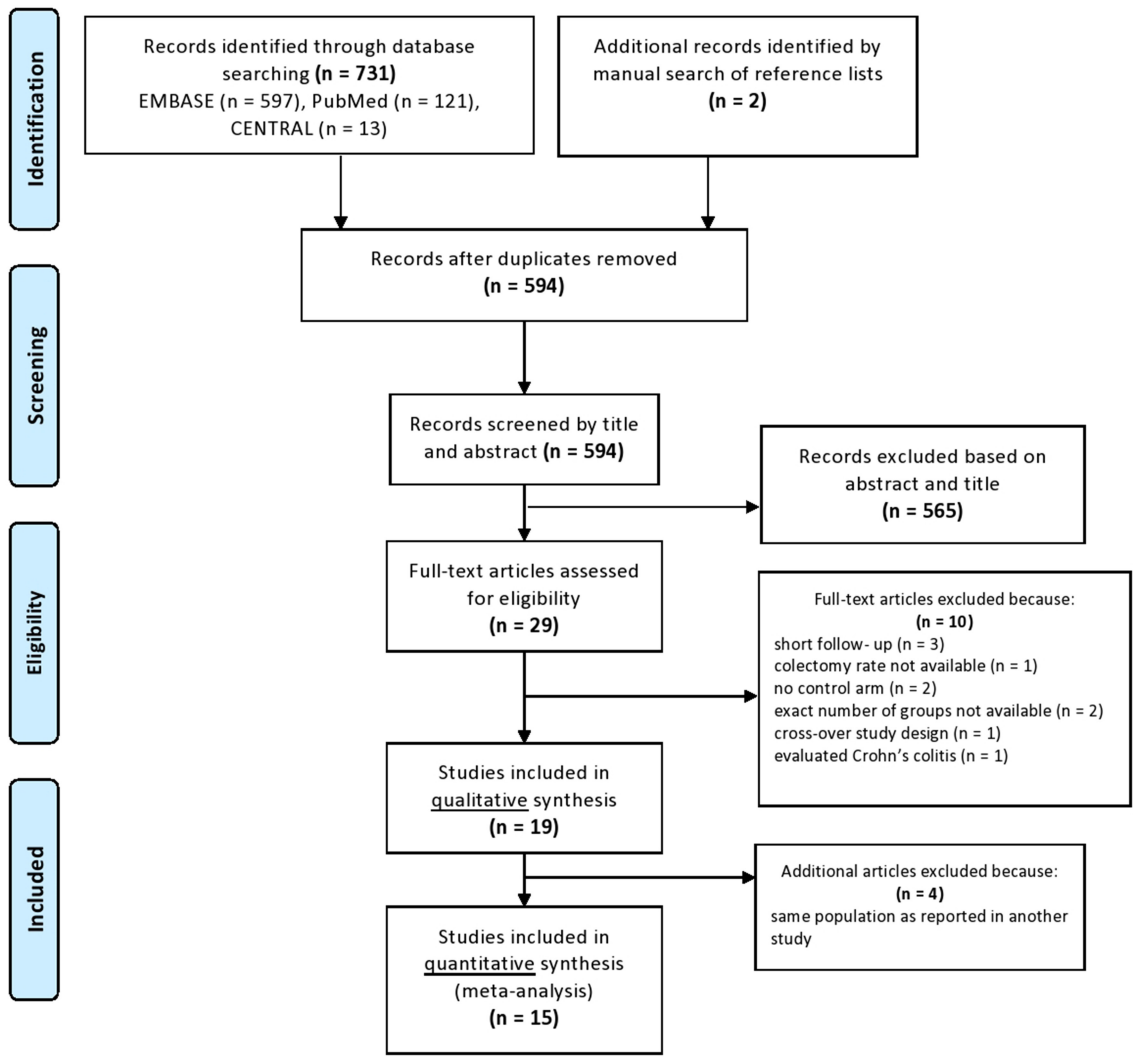
PRISMA flowchart.

A total of 731 records were identified from the databases with our systematic search strategy (121 records in PubMed, 597 ones in EMBASE and 13 ones in CENTRAL) (as shown in the PRISMA flow diagram; Figure 4). Two additional articles were found from the reference lists of the included studies (19, 38). After the removal of duplicates, 594 records remained, 565 of which were excluded by titles and abstracts. The remaining 29 articles were assessed for eligibility by full-text and further 10 studies were excluded due to the following reasons: three studies reported only short-term follow-up data (39–41), one study did not report on the timing and rate of colectomy (38) and two studies were uncontrolled (42, 43). In two studies, the number of patients treated with CYS and IFX was not reported (44, 45), one study included patients pre-treated with either CYS or IFX (46) and one study evaluated patients with Crohn's colitis (47). Nineteen studies remained, but four additional studies were excluded from the quantitative synthesis because they investigated overlapping study population (12, 48–50). Thus, 15 studies fulfilled all inclusion criteria and were included in the meta-analysis.

### Characteristics of the Studies Included

The main characteristics of the included studies are listed in Table 1. The studies were published between 2004 and 2018 and the follow-up period ranged at least from 1 year to maximum of 10 years. In the quantitative analysis, we used data from three RCTs (13, 18, 19) and 12 cohort studies (20–31). A total number of 1,607 patients with steroid-refractory ASUC were included, 879 of which (54.7%) were treated with CYS and the other 728 (45.3%) with IFX. The most common definitions of ASUC used in the studies were the Truelove and Witts criteria, the Mayo and the Lichtiger scores (7, 51, 52). Three of the 15 articles were published only in conference abstract form (19, 29, 31).

In most of the studies, the standard 2 mg/kg/day IV CYS regimen was applied, oral CYS was used for induction of remission only in two studies (19, 21). After the oral CYS bridging, AZA maintenance therapy was continued in all studies. Standard 5 mg/kg dose of IFX was administered in multiple IV infusions (at 0, 2, and 6 weeks) following the induction protocol. Only two studies reported a single infusion of IFX (20, 30). In the IFX treatment groups, AZA was the most commonly administered maintenance drug, albeit recent studies continued IFX (13, 23, 24, 26, 28). Due to the lack of available safety data during long-term follow-up in an RCT, the CYSIF trial (18), AE and SAE results reported in the original study were used in the meta-analysis (12).

### Long-Term Colectomy-Free Survival

**Figure 5:**
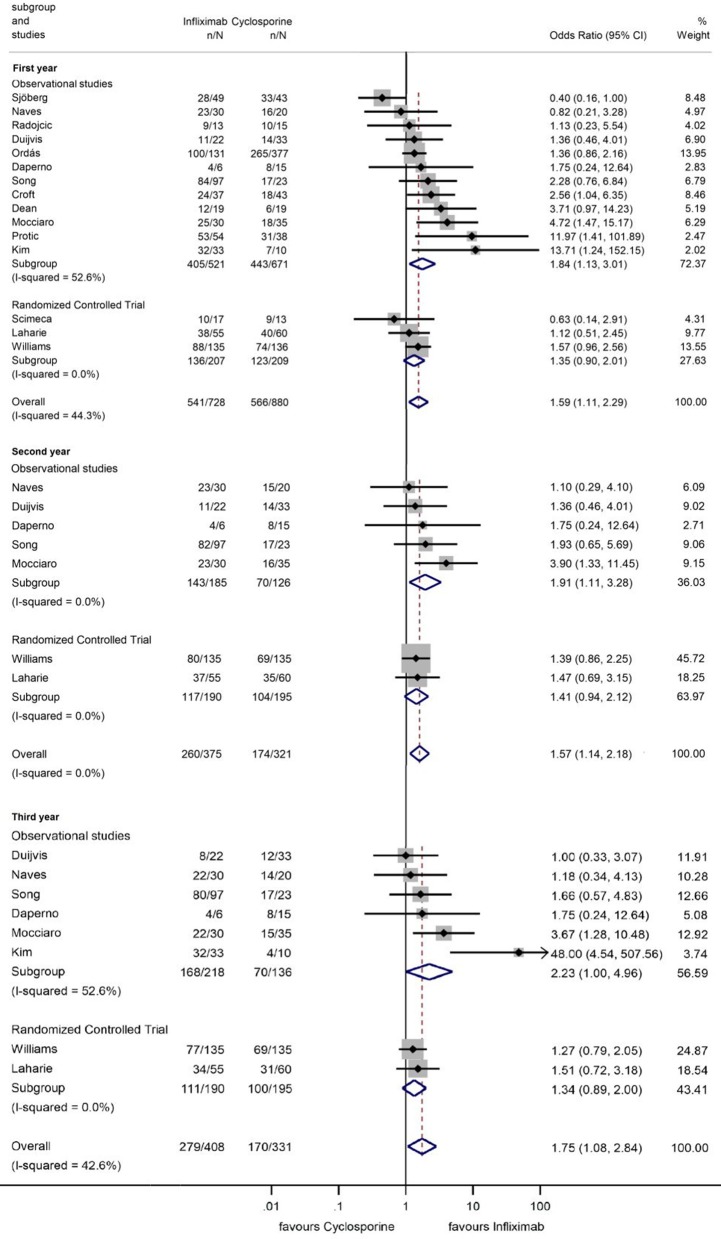
Odds ratios of colectomy-free survival with infliximab (vs. cyclosporine) in the first, second, and third year in steroid-refractory acute severe ulcerative colitis.

**Figure 6:**
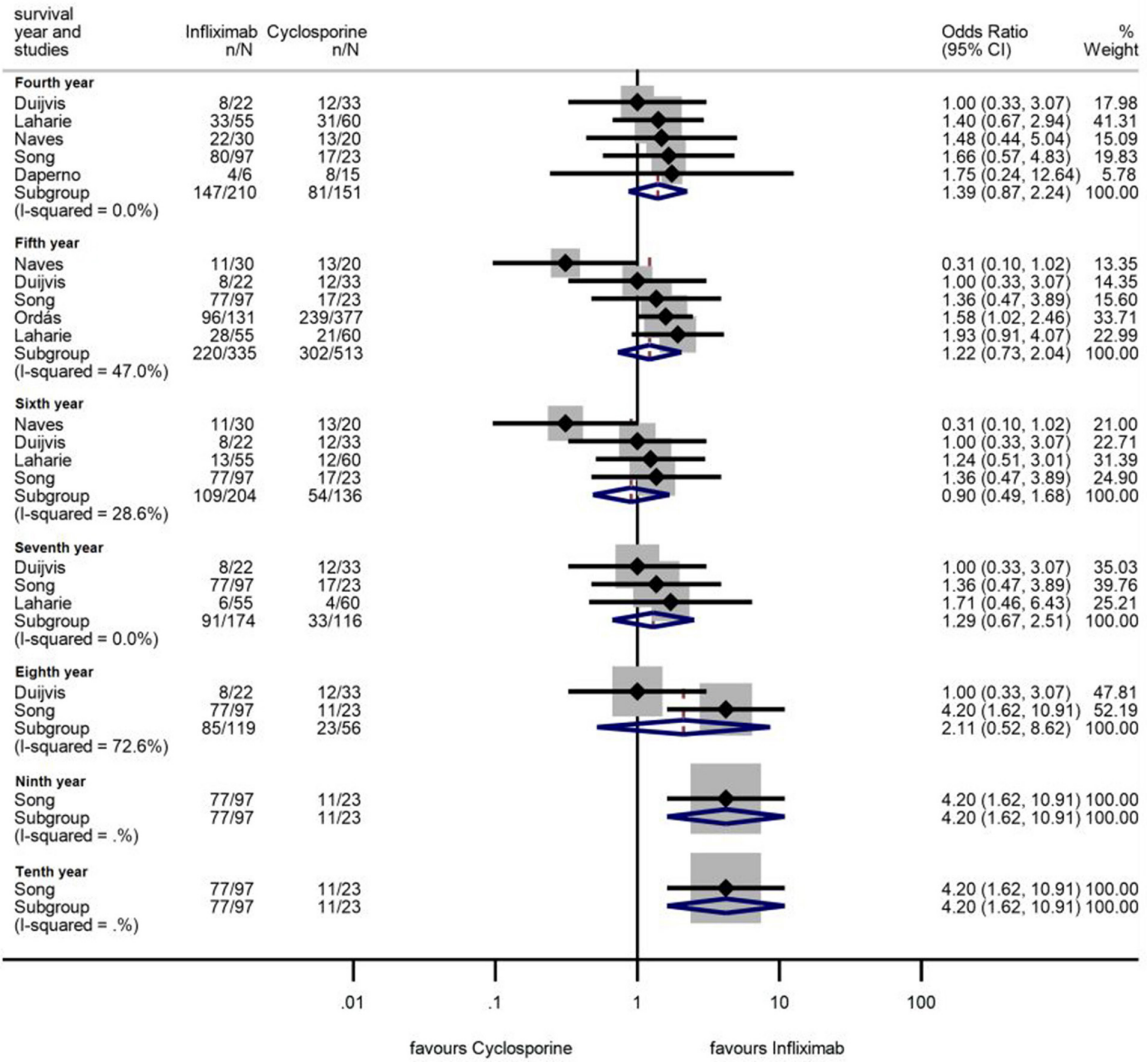
Odds ratios of colectomy-free survival with infliximab (vs. cyclosporine) between the fourth and tenth year in steroid-refractory acute severe ulcerative colitis.

Fifteen, eight, five, and one studies reported 1, 3, 5, and 10 years colectomy-free survival rate. In the first 3 years, colectomy-free survival rate was higher with IFX compared to that with CYS (OR = 1.59, 95% CI: 1.11 −2.29, *p* = 0.012 for 1 year; OR = 1.57, 95% CI: 1.14–2.18, *p* = 0.006 for 2 years; and OR = 1.75, 95% CI: 1.08–2.84, *p* = 0.024 for 3 years), with moderate heterogeneity across the studies (*I*^2^ = 44.3%, *p* = 0.033; *I*^2^ = 0.0%, *p* = 0.74, and *I*^2^ = 42.6%, *p* = 0.093, respectively) (Figure 5). From the fourth year of follow-up, no significant difference regarding the colectomy-free rates was found between the two treatment groups (Figure 6). At 9 and 10 years of follow-up, only one small, retrospective study remained in the analysis, where the colectomy-free survival was higher with IFX compared to that with CYS (31).

However, separating the data of RCTs revealed that the significant association can only be seen if observational studies are included (ORs for observational studies = 1.84, 95% CI: 1.13–3.01, *p* = 0.015 in the first year; OR = 1.91, 95% CI: 1.11–3.28, *p* = 0.020 in the second year; and OR = 2.23, 95% CI: 1.00–4.96, *p* = 0.049 in the third year; ORs for RCTs = 1.35, 95% CI: 0.90–2.01, *p* = 0.143 in the first year; OR = 1.41, 95% CI: 0.94–2.12, *p* = 0.096 in the second year; and OR = 1.34, 95% CI: 0.89–2.00, *p* = 0.157 in the third year) (Figure 5). The heterogeneity remained substantial in the analysis from observational studies but was negligible if RCTs were included exclusively (in the first year *I*^2^ = 52.6%, *p* = 0.016 and *I*^2^ = 0.0%, *p* = 0.466, respectively). TSA indicated that the analysis on colectomy-free survival at 1 year was underpowered, since the monitoring boundaries were not crossed, and the required information size was not reached (Figure 1). According to TSA, at least 1,502 patients would be required for drawing final conclusion while only 416 patients were included in the current analysis.

Based on our strict and consistent grading, the quality of the evidence for colectomy-free survival rates at 1, 3, 5, and 10 years proved to be low for the subgroups of RCTs and very low if non-randomized studies were included as well (Table 3).

**Table 3:** Investigation of quality of the evidence for all included outcomes (GRADE).

**Measured outcomes**	**Study design**	**Risk of bias**	**Inconsistency**	**Indirectness**	**Imprecision**	**Publication bias**	Other (upgrading factors^*^)	**Quality of the evidence**
Colectomy-free rate in the first year	Non-randomized studies (*n* = 12) (starts as low quality)	Data are from studies at low risk of bias	Low heterogeneity (*I*^2^ = 0%)	Evidence that the studies found is no more restrictive then our PICO	Small sample sizes (<400 patients) (−1)	All results come from small studies (−1)	None	Very low ^•^°°°
	RCTs (*n* = 3) (starts as high quality)	Data are from studies at low/unclear risk of bias	Low heterogeneity (*I*^2^ = 0%)	Evidence that the studies found is no more restrictive then our PICO	Small sample sizes (<400 patients) (−1)	All results come from small studies (−1)	None	Low ^•^^•^°°
Colectomy-free rate in the third year	Non-randomized studies (*n* = 6) (starts as low quality)	Data are from studies at low risk of bias	Moderate heterogeneity (*I*^2^ > 60%) (−1)	Evidence that the studies found is no more restrictive then our PICO	Small sample sizes (<400 patients) (−1)	All results come from small studies (−1)	None	Very low ^•^°°°
	RCTs (*n* = 2) (starts as high quality)	Data are from studies at low risk of bias	Low heterogeneity (*I*^2^ = 0%)	Evidence that the studies found is no more restrictive then our PICO	Small sample sizes (<400 patients) (−1)	All results come from small studies (−1)	None	Low ^•^^•^°°
Colectomy-free rate in the fifth year	Non-randomized studies (*n* = 4) (starts as low quality)	Data are from studies at low risk of bias	Moderate heterogeneity (*I*^2^ > 60%) (−1)	Evidence that the studies found is no more restrictive then our PICO	Small sample sizes (<400 patients) (−1)	All results come from small studies (−1)	None	Very low ^•^°°°
	RCTs (*n* = 1) (starts as high quality)	Data are from studies at low risk of bias		Evidence that the studies found is no more restrictive then our PICO	Small sample sizes (<400 patients) (−1)	All results come from small studies (−1)	None	Low ^•^^•^°°
Colectomy-free rate in the tenth year	Non-randomized studies (*n* = 1) (starts as low quality)	Data are from studies at low risk of bias	NA	Evidence that the studies found is no more restrictive then our PICO	Small sample sizes (<400 patients) (−1)	All results come from small studies (−1)	None	Very low ^•^°°°
	(No RCT in the tenth year)	NA	NA	NA	NA	NA	NA	NA
Adverse events	Non-randomized studies (*n* = 5) (starts as low quality)	Data are from studies at low/high risk of bias (−1)	Moderate heterogeneity (*I*^2^ >60%) (−1)	Evidence that the studies found is no more restrictive then our PICO	Small sample sizes (<400 patients) (−1)	All results come from small studies (−1)	None	Very low ^•^°°°
	RCTs (*n* = 2) (starts as high quality)	Data are from studies at low risk of bias	Low heterogeneity (*I*^2^ = 0%)	Evidence that the studies found is no more restrictive then our PICO	Small sample sizes (<400 patients) (−1)	All results come from small studies (−1)	None	Low ^•^^•^°°
Serious adverse events	Non-randomized studies (*n* = 5) (starts as low quality)	Data are from studies at low/unclear risk of bias (−1)	Low heterogeneity (*I*^2^ = 0%)	Evidence that the studies found is no more restrictive then our PICO	Small sample sizes (<400 patients) (−1)	All results come from small studies (−1)	None	Very low ^•^°°°
	RCTs (*n* = 3) (starts as high quality)	Data are from studies at low/unclear risk of bias (−1)	Low heterogeneity (*I*^2^ = 0%)	Evidence that the studies found is no more restrictive then our PICO	Small sample sizes (<400 patients) (−1)	All results come from small studies (−1)	None	Low ^•^^•^°°
Mortality	Non-randomized studies (*n* = 2) (starts as low quality)	Data are from studies at low/high risk of bias (−1)	Low heterogeneity (*I*^2^ = 0%)	Evidence that the studies found is no more restrictive then our PICO	Small sample sizes (<400 patients) (−1)	All results come from small studies (−1)	None	Very low ^•^°°°
	RCTs (*n* = 3) (starts as high quality)	Data are from studies at low/unclear risk of bias (−1)	Low heterogeneity (*I*^2^ >40%)	Evidence that the studies found is no more restrictive then our PICO	Small sample sizes (<400 patients) (−1)	All results come from small studies (−1)	None	Low ^•^^•^°°

* *Including large effect, dose response, no plausible confounding factors, NA, non-applicable*.

### Safety

**Figure 7:**
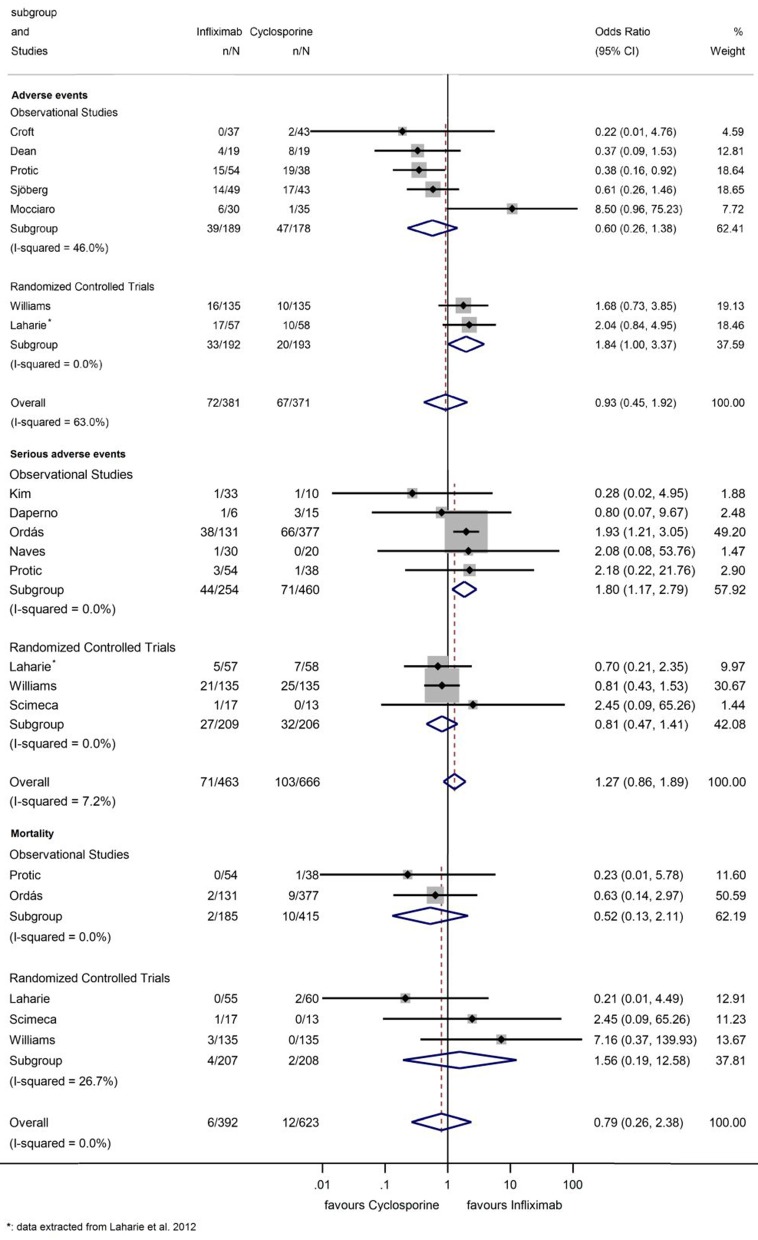
Odds ratios of studies evaluating adverse events, serious adverse events, and mortality during infliximab treatment compared to the cyclosporine group in steroid-refractory acute severe ulcerative colitis.

Seven studies assessed AE (Figure 7) (12, 13, 20, 22, 25, 28, 30). Sixty-seven (18.1%) AEs were reported with CYS and 72 (18.9%) with IFX. The pooled OR of AEs was 0.93 (95% CI: 0.45–1.92, *p* = 0.847), demonstrating no significant difference between groups (Figure 7).

The cumulative Z-curve of the risk of AE during TSA reached but not crossed the conventional boundary (Figure 2). The number of participants included (*n* = 385) did not reach the required information size (*n* = 749), the cumulative Z-curve does not cross the monitoring boundary either.

Eight studies reported on SAE, such as opportunistic infections, sepsis, anaphylactic reaction and hepato- and nephrotoxicity (Figure 7) (12, 13, 19, 21, 24, 26–28). One hundred and three (15.5%) SAEs were reported with CYS and 72 (15.3%) with IFX. Rate of SAE was not elevated with IFX compared to that with CYS (OR = 1.27, 95% CI: 0.86–1.89, *p* = 0.236); although in the subgroup analysis of observational studies (21, 24, 26–28), IFX was associated with a higher SAE rate (OR = 1.80, 95% CI: 1.17–2.79, *p* = 0.008). However, in the three RCTs (13, 18, 19), no statistically significant difference could be detected between the two groups (OR = 0.81, 95% CI: 0.47–1.41, *p* = 0.461), data proved to be homogeneous (*I*^2^ = 0.0%, *p* = 0.712; *I*^2^ = 0.0%, *p* = 0.781, and *I*^2^ = 7.2%, *p* = 0.374 for observational and randomized studies and overall, respectively).

TSA of SAE showed that the number of patients in the analysis of RCTs did not reach the required information size and the cumulative Z-curve did not cross the monitoring boundary (Figure 3).

There was also no significant difference between treatment groups regarding mortality (OR: 0.79, 95% CI: 0.26–2.38, *p* = 0.678; *I*^2^ = 0.0%, *p* = 0.411) (Figure 7) (13, 18, 19, 27, 28).

The GRADE assessment of safety outcomes (AE, SAE, and mortality) showed low quality of evidence for the analyses of RCTs and very low quality of evidence for that of non-randomized studies (Table 3).

### Risk of Bias Assessment

**Figure 8:**
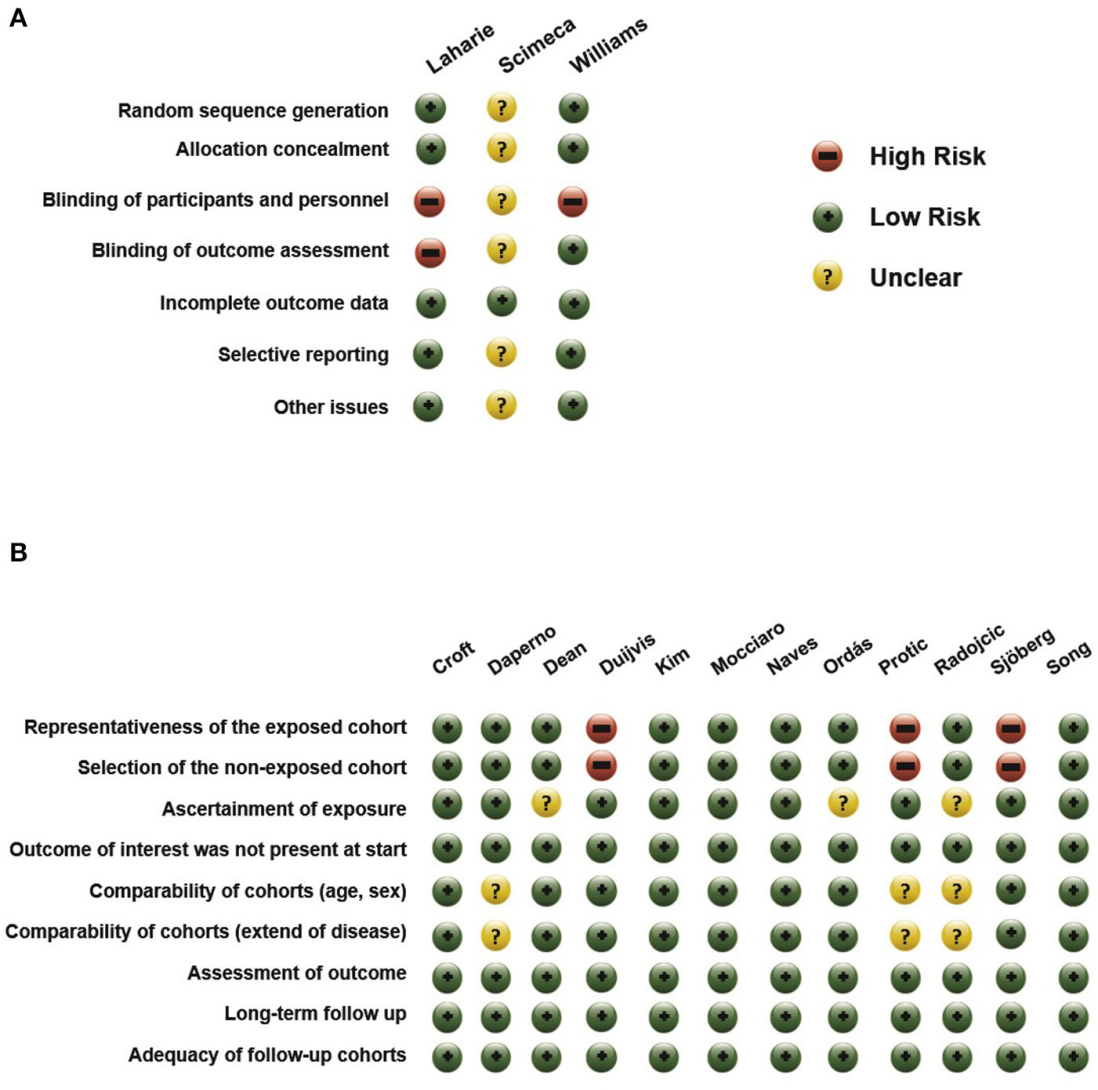
Risk of bias in **(A)** RCTs and in **(B)** non-randomized studies.

Assessments of the risk of bias of the included studies are shown in Figure 8. In the observational studies, the representativeness of the exposed and the selection of the non-exposed cohort was judged to be at high risk in multiple studies (23, 28, 30). In the studies of Daperno, Protic, and Radojcic, no comparison was performed between groups regarding age, sex, and extent of disease (21, 28, 29).

Among the RCTs, the studies of Williams and Laharie carried the lowest risk of bias (13, 18). As they were open trials, participants and personnel were not blinded; however, in the study of Williams, outcome assessment remained blinded. Because the study of Scimeca et al., was only published in a conference abstract form, almost all domains were judged as carrying “unclear” risk of bias (19).

## Discussion

### Summary of Main Findings

ASUC is a medical emergency and should be managed in high-volume tertiary centers with a multidisciplinary approach. In patients failing to respond to parenteral corticosteroids, medical rescue therapy including CYS or IFX is needed. Recently, a meta-analysis has covered the short-term efficacy of the two drugs in treatment response and 12 months colectomy rates but failed to discuss long-term outcomes (14).

In our meta-analysis, we collected RCTs and observational studies to perform long-term statistics focusing on colectomy-free survival rates and drug safety. Our combined data from all the studies showed that there was a significantly higher colectomy-free survival rate with IFX compared to that with CYS. This difference was only seen within the first 3 years after rescue therapy was initiated and it disappeared after the fourth year of follow-up. Additionally, we performed a subgroup analysis by study design to reveal selection bias when comparing RCTs and observational studies. Higher colectomy-free survival was found in observational studies with IFX but not in RCTs. It should be noted that the level of heterogeneity was moderate to substantial in the analysis from observational studies whilst data from RCTs were homogenous. When evaluating safety outcomes, no significant difference was detected between CYS and IFX treatment groups regarding AE, SAE, and mortality. The neutral association calculated from RCTs proved to be underpowered (indicated by TSA) and therefore insufficient to draw a final conclusion (36).

During ASUC treatment, early identification of steroid refractoriness and early introduction of rescue treatments are crucial to avoid morbidity and mortality. A variety of risk prediction tools have been developed to identify patients with ASUC being suitable for second-line medical therapy, these tools are used in clinical practice (such as the Oxford criteria and the Ho index) (53, 54). In a retrospective study, older age, severe endoscopic lesions, high CRP, low albumin levels and low serum IFX levels were identified as predictors of IFX failure in ASUC (30). Due to increased intestinal loss of IFX in ASUC, the serum IFX levels could be decreased; therefore, a modified IFX induction strategy can be considered (55). However, the results of other studies have opposed this association. Dose optimization based on IFX drug level monitoring may result in better patient outcomes (56, 57). In a retrospective study and meta-analysis, no association was found between accelerated IFX induction therapy and lower rates of colectomy in patients with ASUC, compared to standard induction therapy (58). The benefit of intensified induction regimen, i.e., shorter dosing intervals and/or higher doses of IFX is still not proven.

### Limitations and Strengths

However, we are aware that our findings suffer from several limitations. First, most of the studies were non-randomized, retrospective studies, and the number of RCTs was low. Second, the use of maintenance therapy after the initial response was not uniform in all studies and must have contributed to the variation in the long-term outcomes. Third, the definitions of AE and SAE were often mixed together and were unclear in the reports; therefore, an internationally accepted guide has been adopted (17). Fourth, in two RCTs (13, 18), there is switch reported in some cases between CYS and IFX or IFX and CYS as third-line rescue therapy. The switch was necessary to avoid colectomy and achieve clinical remission. However, this can cause a difficulty defining the effect of the drug and may affect the long-term outcome.

We deviated from the PROSPERO protocol regarding an important point. Originally, the primary outcome was planned to be the 5 years colectomy-free survival. However, we thought that investigating the same outcome at multiple time points may improve the clinical yield of!!break the results.

Last, conference abstracts with limited information were also included in the meta-analysis, containing a high amount of unclear information and an increasing possibility of risk of bias.

There are several strengths of our meta-analysis that worth being highlighted. Altogether, a high number of patients with ASUC was investigated. Our meta-analysis is the first reporting more than 1 year colectomy free-survival rates with a high number of studies providing even seven or 10 years of colectomy-free survival data. Another strength of our meta-analysis is that the certainty of the evidence was examined for all outcomes according to the GRADE approach (37). Moreover, TSA was used to test whether the analyses are sufficiently powered; therefore, can be considered conclusive.

## Conclusions

In summary, our meta-analysis has shown that there is no definitive evidence for any difference regarding long-term efficacy and safety between CYS and IFX in patients with steroid-refractory ASUC based on RCTs. Considering second-line treatment options in ASUC, the choice of drug depends on several factors other than efficacy and safety. Since the introduction of IFX, as rescue therapy for ASUC and a proxy for CYS, the length of hospital stay and in-hospital costs have been reduced significantly (59). On the other hand, the total costs up to 3 months after initiation of rescue therapy were significantly higher in the IFX group (59). However, since 2013 lower-cost IFX biosimilars are available, which may result in large cost savings in the future. In addition to safety and efficacy, other components of evidence-based medicine, such as the experience of treating physicians and patient preferences, should also be highlighted. In thiopurine-naïve patients, CYS can be preferred as a bridge to thiopurine maintenance treatment. IFX is a reasonable option for patients who have previously failed thiopurine maintenance therapy. Results of TSA and the lack of high-quality evidence in our meta-analysis highlight that further large RCTs are warranted to decide which therapy is the preferable rescue therapy in ASUC.

## Data Availability Statement

The datasets analyzed in this article are not publicly available. Requests to access the datasets should be directed to KS, szemesk@gmail.com.

## Author Contributions

PS and KS designed the research. PS, KS, NF, and AS performed the research and statistical analyses, analyzed and interpreted the data. KS and PS wrote the article. BE, EM, KM, AE, and ZS made the critical revisions related to important intellectual content of the manuscript. PS and PH gave the final approval of the version of the article to be published.

### Conflict of Interest

The authors declare that the research was conducted in the absence of any commercial or financial relationships that could be construed as a potential conflict of interest.

## Abbreviations

AE: adverse events
ASUC: acute severe ulcerative colitis
AZA: azathioprine
CRP: C-reactive protein
CYS: cyclosporine
ESR: erythrocyte sedimentation rate
IFX: infliximab
IV: intravenous
OR: odds ratio
RCT: randomized controlled trial
SAE: serious adverse events
TNFα: tumor necrosis factor-alpha
TSA: trial sequential analysis
UC: ulcerative colitis.
